# Cytochrome c-554 from *Methylosinus trichosporium* OB3b; a Protein That Belongs to the Cytochrome c2 Family and Exhibits a HALS-Type EPR Signal

**DOI:** 10.1371/journal.pone.0022014

**Published:** 2011-07-18

**Authors:** Espen Harbitz, K. Kristoffer Andersson

**Affiliations:** Department of Molecular Biosciences, University of Oslo, Oslo, Norway; Griffith University, Australia

## Abstract

A small soluble cytochrome *c*-554 purified from *Methylosinus trichosporium* OB3b has been purified and analyzed by amino acid sequencing, mass spectrometry, visible, CD and EPR spectroscopies. It is found to be a mono heme protein with a characteristic cytochrome *c* fold, thus fitting into the class of cytochrome *c*_2_, which is the bacterial homologue of mitochondrial cytochrome *c*. The heme iron has a Histidine/Methionine axial ligation and exhibits a highly anisotropic/axial low spin (HALS) EPR signal, with a *g*_max_ at 3.40, and ligand field parameters V/ξ  = 0.99, Δ/ξ  = 4.57. This gives the rhombicity V/Δ  = 0.22. The structural basis for this HALS EPR signal in Histidine/Methionine ligated hemes is not resolved. The ligand field parameters observed for cytochrome *c*-554 fits the observed pattern for other cytochromes with similar ligation and EPR behaviour.

## Introduction

The bacterium *Methylosinus trichosporium* OB3b is an obligatory methanotrophic gram-negative bacterium that can utilize methane as its only source of carbon and energy [1], [2], and is one of the most studied methanotrophic alphaproteobacteria. In addition to be able to oxidize methane, *Methylosinus trichosporium* Ob3b is able to oxidize a broad spectrum of toxic and organic compounds otherwise difficult to degrade, including halogenated hydrocarbons as trichloroethylene [3], [4]. This ability to oxidize potential biocides has led to an interest in using *Methylosinus trichosporium* OB3b for bioremediation of recalcitrant organic pollutants. *Methylosinus trichosporium* OB3b have two different enzyme systems capable of oxidizing methane, the soluble methane monooxygenase (sMMO) [5] and the membrane-bound particulate methane monooxygenase (pMMO) [6]. The expression of these enzyme systems (relative to each other) varies with the growth conditions, most importantly the copper concentration. High concentrations of copper(II) results in a high proportion of pMMO, while sMMO dominates at low copper(II) concentrations [2], [5], [6], [7]. In bacterial methane metabolism, a soluble *c*-type cytochrome is known to be involved in the oxidation of methanol to methanal by methanol dehydrogenase in the periplasmic space [2], [8].

*C*-type cytochromes are a diverse group of electron transfer proteins found in all areas of life. They are known to play important parts in key metabolic processes as respiration and photosynthesis. The covalent attachment of the heme group to the protein backbone is the key characteristic of these proteins and it is suggested that this is important for retaining the heme group outside the cytoplasm or in the mitochondrial intermembrane space [9], but it is proposed that heme attachment influences both heme reduction potential and ligand-iron interaction [10]. This attachment is formed by two (in some cases only one) thioether bonds with cysteine residues. The heme binding motif, Cys-X-X-Cys-His, is highly conserved and provides cysteine residues for the covalent attachment as well as a histidine axial ligand coordinating to the heme iron. The histidine is structurally constrained and the imidazole plane aligned roughly along the heme α-γ meso axis. The other ligand is most commonly methionine (e.g. mitochondrial cytochrome *c* [11], in the photosynthetic reaction centre [12] and soluble bacterial cytochromes [13], [14], [15]), but it can be histidine (e.g. cytochrome *cd*_1_), the N-terminal amino group (cytochrome *f*) [16] or the iron can be five coordinated, as in cytochrome *c*' [17]. Historically different naming systems have been employed for the *c*-type cytochromes according to their physical properties, such as spectral features, redox potential or isoelectric point. In 1982, Ambler identified four major classes of *c*-type cytochromes based mainly on their sequence [18]. More recently Bertini and co-workers have added groups to this list when they scanned 188 genomes for cytochrome *c* domains, in order to obtain a wide coverage of the possible roles of *c*-type cytochromes [19]. The analysis of bacterial genomes revealed an astonishing variety in the number and types of *c*-type cytochromes encoded by different bacteria.

In this study we report purification and properties of a cytochrome *c*-554 from *Methylosinus trichosporium* OB3b, grown under pMMO expressing conditions. We compare this cytochrome with cytochrome *c*-555 from *Methylococcus capsulatus* (Bath), which was purified using the same protocol. We observe that these two cytochromes, both from methane oxidising bacteria, exhibit markedly different ferric low-spin EPR spectra. Cytochrome *c*-554 from *Methylosinus trichosporium* OB3b exhibits a highly anisotropic/axial low spin (HALS) EPR signal, whereas cytochrome *c*-555 from *Methylococcus capsulatus* (Bath) exhibits a rhombic EPR signal. Since the relationship between structure and these EPR signals in His/Met-ligated hemes is not understood, the characterization of more His/Met coordinated heme systems may be helpful in explaining the physical basis of the different EPR spectral types [20].

## Methods

### Materials

Horse heart cytochrome *c* was obtained from Sigma-Aldrich, cytochrome *c*-552 from *Nitrosomonas europaea* was donated by Professor Alan B. Hooper, University of Minnesota [15], [21], and cytochrome *c*-553 from *Bacillus pasteurii* was donated by Professor Stefano Ciurli, University of Bologna [13].

### Bacterial Growth

*Methylosinus trichosporium* OB3b culture was obtained from the laboratory of John D. Lipscomb (University of Minnesota). Fermentation was conducted in a 10 L Biostat bioreactor (B. Braun, Melsungen) using a previously described culture medium [22] containing 10 µM copper sulphate. Cells were grown at 30°C and an agitation rate of 200–300 rpm and were purged with a 1∶4 methane/air mixture. The pH in the fermentor was maintained at 6.8–7.0 by addition of 1 M H_2_SO_4_ (aq). Cells were harvested at an OD_540_ between 3.0 and 5.0. The bacterial culture was first cooled to 0°C using crushed ice. The cooled cell culture was filtered through a Millipore Masterflex Cross-flow filtration unit (45 µm) until the volume was 1 litre. Harvested cells were centrifuged at 7000 g for 10 minutes, washed in 50 mM Pipes buffer, pH 7.0, frozen in liquid nitrogen and stored at −80°C.

### Protein purification of cytochrome *c*-554 from *Methylosinus trichosporium* OB3b

While still in the frozen state, 30 g cells were packed into the cooled **X**-**press**® (AB **BIOX**, Gothenburg) pressure cell [23]. Cells were disrupted by several passages through the 1 mm diameter orifice at a pressure of ∼500 bars. This breaks the ice crystals irrespective of the cell membranes, thus disrupting the cells. The pressate was thawed and diluted with 120 ml 10 mM Tris HCl buffer, pH 8.2. The suspension was incubated with DNase I solution (80 Kunitz units) for 60 minutes at 4°C, and homogenized and centrifuged at 12.000 g for 20 minutes and then at 75.000 g for 3 hours. Ammonium sulphate was dissolved in the supernatant until the solution was 75% saturated, stirred for 60 minutes at 4°C, and centrifuged at 13.000 g for 30 minutes. The supernatant was applied to a phenyl sepharose column (1.5 cm×30 cm), equilibrated with 1.5 M (NH_4_)_2_SO_4_ in 10 mM Tris HCl buffer (pH 8.2). The protein was eluted with a linear gradient from 1.5 M to 0 M (NH_4_)_2_SO_4_. The eluted fractions with significant absorption at 410 nm were collected and concentrated in an Amicon® ultrafiltration cell (10 kDa filter cut-off). The ionic strength of the sample was decreased using a NAP- 10 column (GE Healthcare), which is a standardized Sephadex G-25 gel filtration column, before it was applied to a CM sepharose column (1.5 cm×20 cm), which had been equilibrated with 10 mM Tris HCl buffer (pH 8.2). The protein was eluted with a linear gradient from 0 to 0.15 M KCl in 10 mM Tris HCl buffer (pH 8.2), and then concentrated. The protein solution was applied to a Sephadex G-75 gel filtration column (1.5 cm×30 cm) equilibrated with 10 mM Tris HCl buffer (pH 8.2), concentrated and stored at −80°C. The same procedure was used to purify cytochrome *c*-555 from *Methylococcus capsulatus* (Bath).

### Protein Sequencing

Protein sequencing was performed by automatic Edman degradation on an Applied Biosystems 477A Protein Sequencer (Applied Biosystems, Foster City, CA, USA) with an online 120A PTH amino acid analyser in the lab of Knut Sletten [24]. Cyanogen bromide (CNBr) cleavage of the protein was performed by dissolving 50 nmol freeze dried protein in 70% formic acid followed by an addition of a 50-fold excess of CNBr [25]. The mixture was left to incubate in darkness at 20°C for 24 hours. The CNBr was removed by evaporation before sequencing. The peptide sequences obtained was compared with the newly released genome [26] using TBLASTN, which searches a translated nucleotide sequence using a protein query [27]. The full peptide sequence identified was modelled on the structure peptide backbone of ferrocytochrome *c*_2_ from *Rhodopseudomonas viridis* (1CO6.pdb) using SWISS-MODEL [28]. The protein sequence data reported in this paper will appear in the UniProt Knowledgebase under the accession number D5QVH0.

### EPR Spectroscopy

The EPR spectra were acquired with a Bruker Elexsys 300E 10/12 X–band spectrometer equipped with a Bruker ER4116DM dual mode resonator cavity. A HeFlow cryostat (ESR 900, Oxford Instruments) allowed temperature regulation of the sample in the region 3.6 K to 100 K. The EPR samples were prepared by employing fast freezing procedures (from 273 K to77 K) of the protein solutions placed in EPR tubes. In all the measurements for cytochromes *c*, the EPR signals were too broad to detect above 30 K; thus most of the EPR spectra were collected at 10 K. The experimental conditions was: 9.65 GHz, modulation amplitude 1 mT, modulation frequency 100 kHz, sweep time168 s, time constant 82 ms, 4×10^4^ gain, and the spectra was recorded in perpendicular mode. The spectra were recorded at 10±0.5 K, except for the temperature behaviour studies were data were recorded at 7, 13, 16, 21, 26, 31 and 36 K. The EPR spectra was recorded under non-saturating microwave power, with the exception of the microwave power saturation studies performed at 0.08, 0.2, 0.4, 0.8, 2.01, 6.35, 15.9, 63.5 and 159 mW. The spectral features was simulated with WINEPR SimFonia version 1.25 provided by Bruker.

### Light absorption spectroscopy

UV-Vis transitions were observed on a HP8452A Spectrophotometer and a Jasco J-810 Spectropolarimeter. The heme content was quantified using alkaline pyridine hemochrome [29], which gives distinct absorption band, where the extinction coefficients vary only with the type of heme. (For heme *c* the extinction coefficients for the α- and β-bands are ε_551_  = 29.1 mM^−1^ cm^−1^ and ε_522_  = 18.6 mM^−1^ cm^−1^ [29].) A few grains of dithionite were added to the purified cytochrome *c* solution (50 µM) before an equal amount of a stock solution containing 0.15 M NaOH and 2.1 M pyridine was mixed with the protein solution. The heme concentration measured using alkaline pyridine hemochrome [29], was used to calculate extinction coefficients for the initial cytochrome solution.

### Mass spectroscopy and heme cleavage

The protein mass was established using a Voyager-RP DE mass spectrometer (Applied Biosystems, Foster City, CA, USA) in the linear positive ion mode. Acceleration voltage, grid voltage and the delay time between ion production and extraction were adjusted to get optimal peak resolution for each sample. For each spectrum, 100 single scans were accumulated. The matrix, sinapinic acid (3,5-dimethoxy-4-hydroxy cinnamic acid (D-7927, Sigma-Aldrich, St. Louis, MI, USA)) was dissolved to saturation in a 1∶1 (v/v) mixture of acetonitrile and 0,1% (v) aqueous trifluoroacetic acid. Equal volumes of sample and matrix solution were mixed on the sample plate and air dried. All data were calibrated using an external calibration standard mixture (Applied Biosystems, Foster City, CA, USA). The *c*-type hemes was cleaved from the protein by reacting with 2-nitrophenylsulfenylchloride (2-NPS-Cl). It was performed by mixing 80 µl cytochrome *c* solution (20–30 µM) in 50% acetic acid with 40 µl 2-NPS-Cl (4 mM in acetic acid) and incubated for 10 minutes. Removal of 2-NPS from modified cysteine residues were done by adding 80 µl 0.4% mercaptoethanol (in a 10 M Urea solution) and incubated for another 10 minutes before the protein was desalted on a NAP-5 column equilibrated with 10% acetic acid. In the fractions collected after desalting there were an absence of the characteristic heme absorption bands, but a peak at 365 nm was observed confirming the presence of tryptophan residues modified by 2-NPS-Cl [30]. Ferrous and ferric cytochrome were prepared by addition of an excess of sodium dithionite, Na_2_S_2_O_4_, or potassium ferricyanide, K_3_Fe(CN)_6_, to the cytochrome solution, respectively, and excess reagents were removed on a NAP-5 column.

## Results

### Protein purification of cytochrome *c*-554

The purification procedure was monitored with light absorption by observing the ratio A_410_ /A_280_ as a measure of the heme to protein ratio. At the end of the purification procedure, this ratio approached 8. This is a reasonable value for a pure protein sample when compared with our measured extinction coefficient and the amino acids found in the protein sequence. SDS-PAGE revealed no protein impurities and suggested a molecular weight of about 13000 Da (data not shown). This is in good agreement with the mass obtained by mass spectrometry (see below).

### Visible absorption and CD spectra indicates methionine ligation of cytochrome *c*-554

**Figure 1:**
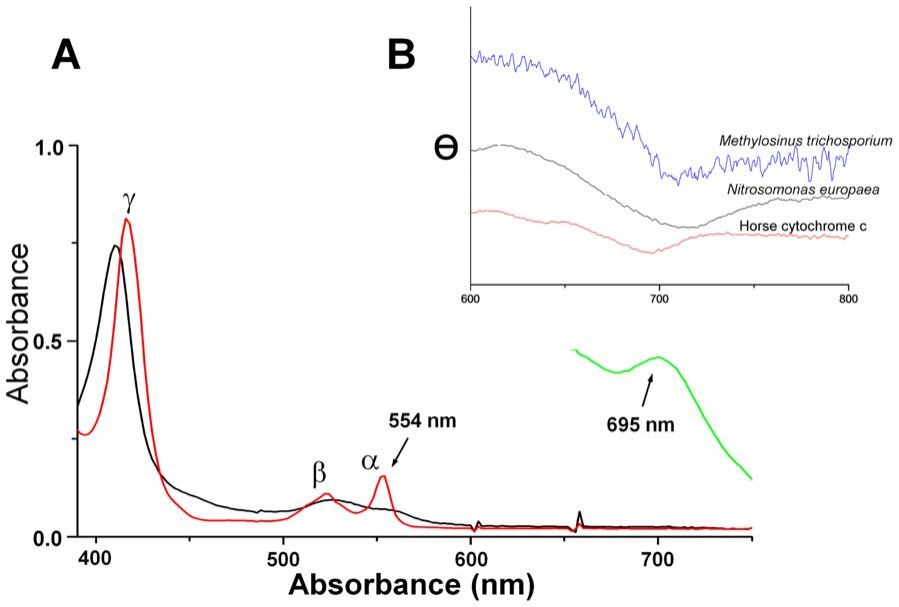
Absorption and CD spectra of cytochrome *c*-554 from *Methylosinus trichosporium* OB3b. A. Visible absorption spectra of a 7 µM solution of the reduced and oxidized cytochrome *c*-554 from *Methylosinus trichosporium* OB3b. The α-band in the reduced spectrum has a maximum at 554 nm used to name the cytochrome. The spectrum of a 83 µM solution of the oxidized protein exhibits a peak at 695 nm in the oxidised spectrum is indicative of methionine ligation to the heme. B. Circular dichroism of a 60 µM solution of cytochrome *c*-554 (magnified 10 times) is compared with a 790 µM solution of cytochrome *c*-552 from *Nitrosomonas europaea* and a 200 µM solution of cytochrome *c* from horse heart. The circular dichroism at the methionine peak shows that the cytochrome *c*-554 has stronger similarities with cytochrome *c*-552 from *Nitrosomonas europaea* than cytochrome *c* from horse heart.

The absorption spectrum of the reduced form the cytochrome had α-, β- and Soret bands at 554, 524 and 416 nm, respectively. Upon oxidation, the Soret band shifted to 410 nm and the β-band to 526 nm (Figure 1A). Heme concentrations were calculated using pyridine hemochrome to obtain an accurate estimate of heme protein content [29]. These protein concentrations were used to calculate the extinction coefficients. The absorption spectrum of the reduced cytochrome has the following extinction coefficients: ε_416_ = 126 mM^−1^ cm^−1^, ε_524_ = 17 mM^−1^ cm^−1^ and ε_554_ = 24 mM^−1^ cm^−1^. The oxidized cytochrome has the extinction coefficients: ε_410_ = 116 mM^−1^ cm^−1^ and ε_526_ = 14 mM^−1^ cm^−1^. The oxidized protein also exhibited a weak band at 695 nm, which is indicative of methionine ligation [31]. The feature at 695 nm was further confirmed by CD spectroscopy [21]. The observed negative CD feature corresponding to this absorption band is red-shifted compared to mammalian cytochrome *c*, similar to what we observe in cytochrome *c*-552 from *Nitrosomonas europaea*, which has the same His/Met heme ligation and a known 3D structure [11], [15] (Figure 1B).

### Mass spectroscopy reveals a single heme group

**Figure 2:**
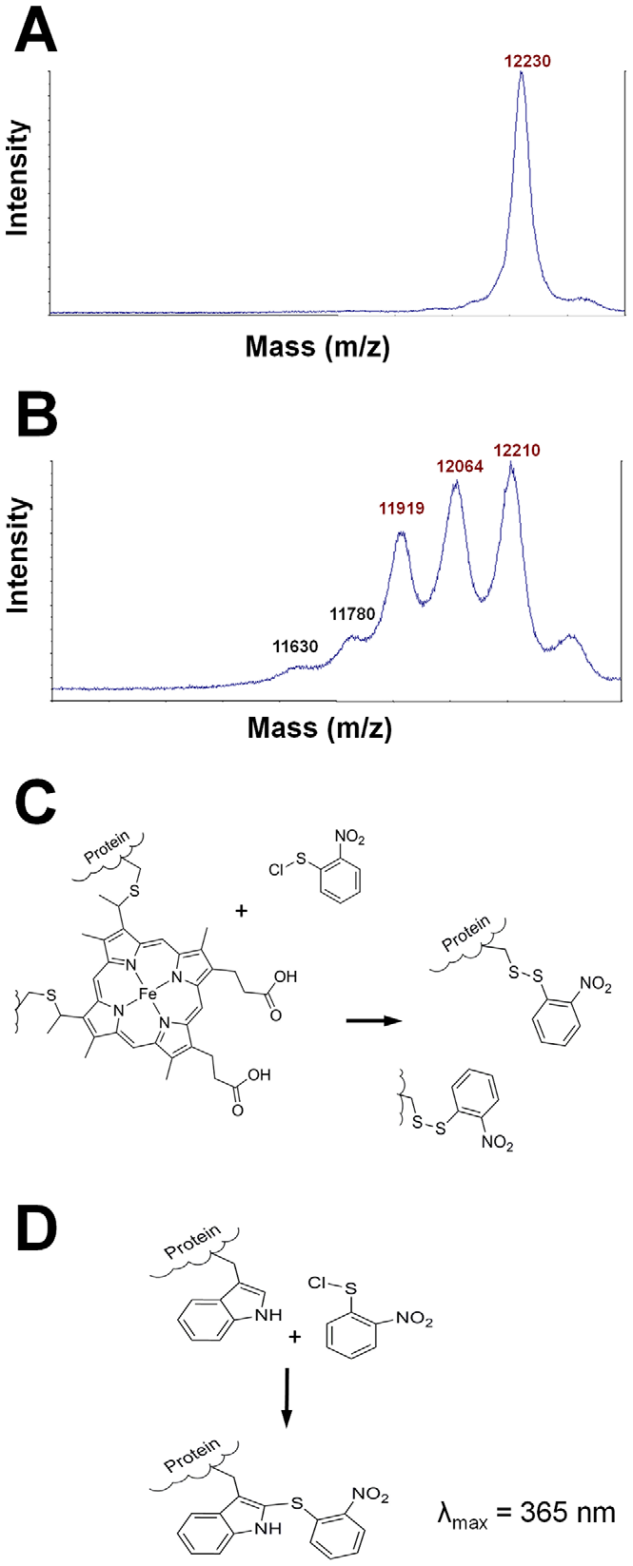
Mass determination and heme cleavage of cytochrome *c*-554 from *Methylosinus trichosporium* OB3b. A. Mass spectrum of the native cytochrome *c*-554 from Methylosinus trichosporium OB3b. B. Mass spectrum of cytochrome *c*-554 after treatment with 2-NPS. The number of peaks is due to protein adducts with different number of added 2-NPS molecules. C. The reaction of 2-NPS with the covalent heme attachment, resulting in a disulphide link between 2-NPS and the protein and loss of the heme group. D. 2-NPS reacts with tryptophan, creating a chromophore with an absorption maximum at 365 nm [30].

There exist many classes of *c*-type cytochromes, with varying number of heme groups attached. Determining the number of hemes attached to a protein might prove difficult, as seen in the case of cytochrome *c*-554 from *Nitrosomonas europaea*, which were believed to contain two hemes but were later found to contain four hemes [32], [33]. To establish the number of heme groups and to obtain an accurate mass determination, MALDI-TOF MS was performed. The molecular weight of the cytochrome was estimated to be 12230±15 Da (Figure 2A). Treatment with 2-nitrophenylsulfenylchloride (2-NPS-Cl) was used to cleave the heme group. The 2-NPS-Cl treated protein exhibited peaks corresponding to a loss of one heme group (Figure 2B). No peaks at lower mass to charge ratios were found, indicating the presence of a single heme group in the protein. Treatment with 2-NPSCl cleaves of the heme group by breaking the thioether bond between the heme group and the cysteine in the CXXCH heme binding motif (Figure 2C). 2-NPS will furthermore react with tryptophan residues to create a chromophore with an absorption maximum at 365 nm (Figure 2D). The observation of this chromophore was used to trace the protein after removal of the heme group, and furthermore suggested the presence of two tryptophan residues in the protein.

### Amino acid sequence of cytochrome *c*-554 from *Methylosinus trichosporium* OB3b

Two parts of the cytochrome *c*-554 from *Methylosinus trichosporium* OB3b were sequenced using Edman degradation [34]. The first 39 amino acids on the N-terminal were determined to be AGDPAAGEKVFNKCKACHQVGETAKNAVAPELNGIDGRK. After cyanogenbromide (CNBr) treatment two peptide fragments were observed, which verified the presence of only one methionine residue in this sequence. The amino acid sequence obtained from the second peptide is MIFPGLSSENDQANVWAYLSQFGADGKK. Sequence alignment indicates that this is the C-terminal part of the protein, and that the methionine at the beginning of the second fragment is the sixth heme axial ligand. The middle part of the protein has not been sequenced. The two sequences, bridged by a gap of unknown length, were aligned using tBlastn with the complete genome of *Methylosinus trichosporium* OB3b [26], [27]. Full identity was found with the bases 13660–13980 in contig00046 of the genome. The complete sequence of the protein contains the following 108 amino acids: H_2_N-AGDPAAGEKVFNKCKACHQVGETAKNAVAPELNGIDGRKSASAEGYNYSEPFKALGITWDEAQFKEFIKNPKAKVPGTKMIFPGLSSENDQANVWAYLSQFGADGKKK-COOH. The heme binding motif and the iron coordinating methionine residue is underlined. The complete protein sequence contains only one heme binding motif and two tryptophans in agreement with the observations from mass spectrometry. There is only one methionine residue in the protein, in position 80, in good agreement with the cyanogenbromide cleavage. The genomic sequence revealed a 24 amino acid signal sequence, MKSISMLTLAASVAFAVTAGQAVA, confirming the periplasmic location of this protein [35]. Based on the primary sequence, a theoretical pI value of 8.55 was calculated using the program ProtParam on the ExPASy server [9], [36].

**Figure 3:**
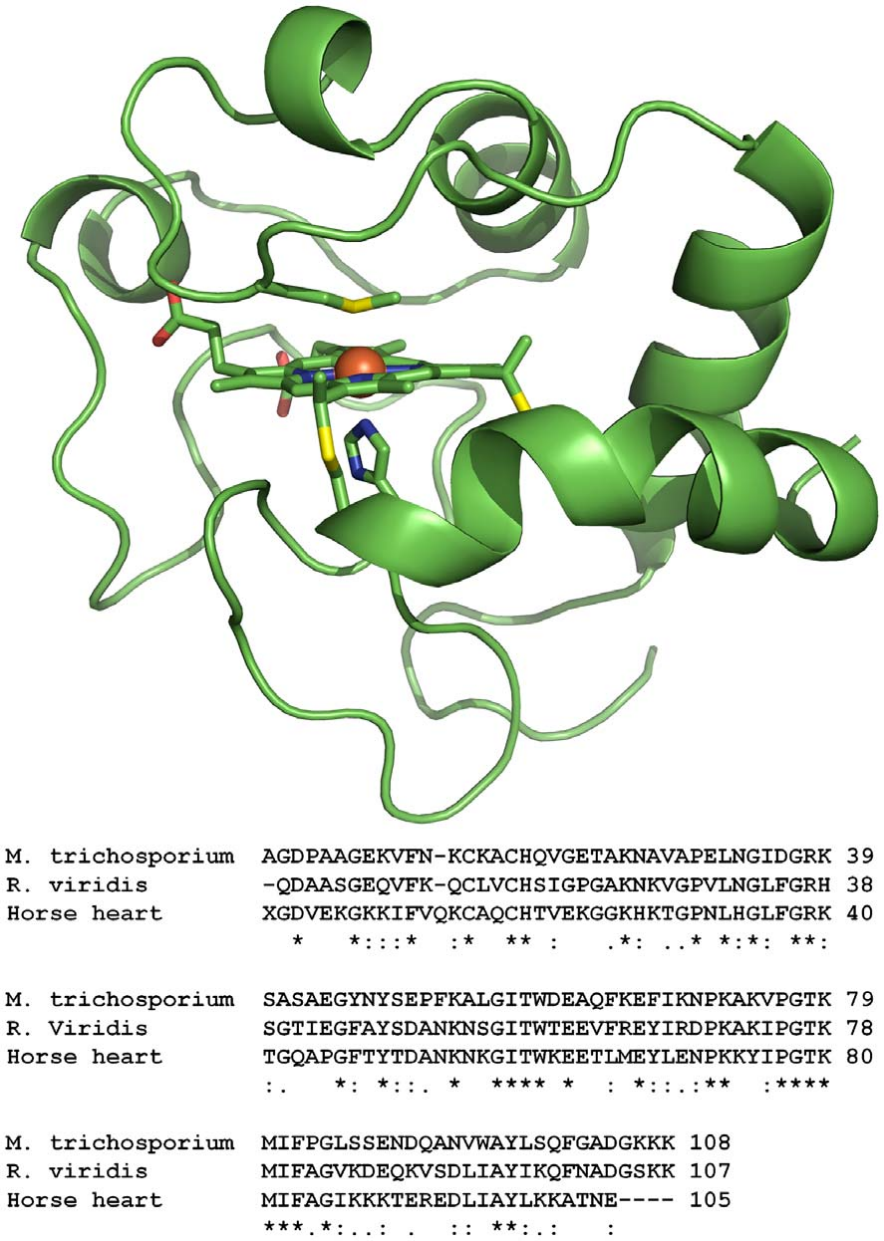
Homology model of cytochrome *c*-554 from *Methylosinus trichosporium* OB3b. The structure template used is cytochrome *c*_2_ from *Rhodopseudomonas viridis* (PDBid: 1CO6) [37]. The amino acid sequence of these two proteins has 56 (52%) identical residues and a total of 74 (69%) positives (identical residues or conservative mutations). High similarity with the mitochondrial cytochrome *c* is found, and sequence alignment reveals a highly conserved region in the loop containing the heme axial ligand methionine. The molecular drawing was made with Pymol, DeLano Scientific, San Carlos, CA.

The sequence was aligned with protein and nucleotide sequence databases using protein blast and tBlastn [27]. The best alignment is found with a *c*-type cytochrome from the genome sequence of *Nitrobacter winogradskyi* strain Nb-255, which has 64% identical residues and 78% positive residues (identical residues or conservative mutations). All best alignments represent *c*-type cytochromes from bacteria belonging to the alphaproteobacteria in the order *Rhizobiales*, in which *Methylosinus trichosporium* OB3b is included. The most similar alignment with a protein with known 3D structure is ferrocytochrome *c*_2_ from *Rhodopseudomonas viridis* with 56 (52%) identical residues, a total of 74 (69%) positive residues and no gaps (pdb-id: 1CO6) [37]. Homology modelling of the tertiary structure using this cytochrome as template reveals a tertiary structure similar to the cytochrome *c* fold of mitochondrial cytochrome *c* (Figure 3) [11], [38]. The identity with horse heart cytochrome *c* is 30.5% (33 out of 108 residues).

### EPR spectroscopy shows that cytochrome *c*-554 exhibits a highly anisotropic low spin (HALS) signal

**Figure 4:**
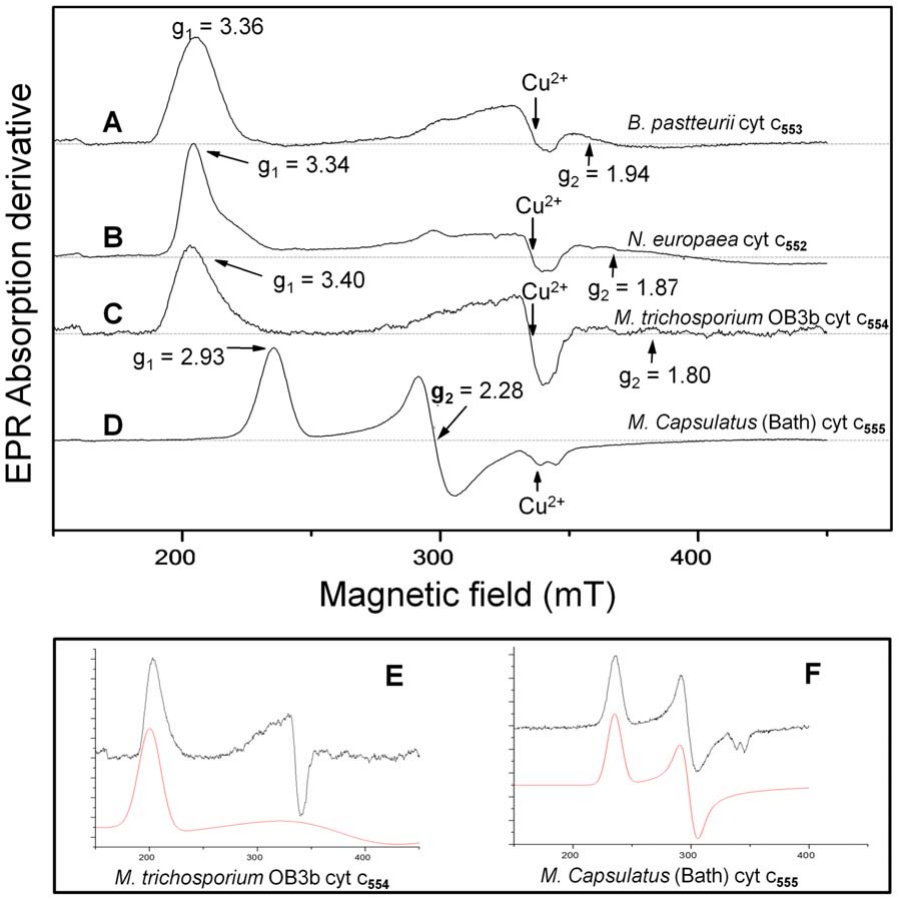
EPR spectroscopy of c-type cytochromes. X-band EPR spectra of cytochromes *c* from: A. *Bacillus pasteurii* (cytochrome *c*-553) [44], B. *Nitrosomonas europaea* (cytochrome *c*-552) [44], C. *Methylosinus trichosporium* OB3b (cytochrome *c*-554), and D. *Methylococcus capsulatus* (Bath) (cytochrome *c*-555). The protein solutions were prepared in 50 mM HEPES buffer at pH 7.5, and the spectra were recorded in perpendicular mode at 10 K with 2 mW microwave power, modulation amplitude 1 mT, modulation frequency 100 kHz, sweep time 168 s, time constant 82 ms and 4×10^4^ gain. E. Simulation of the HALS EPR spectrum C (red). F. Simulation of the rhombic EPR spectrum D (red).

Electron paramagnetic resonance (EPR) spectroscopy probes unpaired electron in a sample. As the only unpaired electrons in proteins occur at metal ions or in free radical enzyme mechanisms, EPR is an excellent tool to study the chemically active part of a protein without interference from the protein backbone. Cytochrome *c*-554 from *Methylosinus trichosporium* OB3b exhibits a ferric HALS (Highly Axial Low-Spin or Highly Anisotropic Low-Spin (S = ½)) also called Type I EPR signal by the nomenclature introduced by F. Ann Walker [39] when subjected to low temperature EPR spectroscopy, with a *g*_max_ value of 3.40 (Figure 4C). HALS EPR signals are also known as “large *g*_max_” signals. This kind of signals have been observed in cytochromes with His/His, His/amine and His/Met coordination of the heme iron, and the main characteristic of these signals is a *g*-value higher than 3.3 [20], [39], [40], [41]. Because of the nature of the very broad HALS EPR signal, direct assignment of all three *g*-values is generally not possible. The microwave absorption amplitude decays slowly and extends to high magnetic fields, which results in a weak slope, and hence small amplitudes in the 1st derivative spectrum. Usually low-spin ferric hemes exhibit rhombic EPR spectra where two distinct *g*-values can be clearly resolved (Figure 4D). In the HALS EPR spectra, the last two *g*-values are very poorly resolved. HALS EPR spectra can be observed in cytochromes *c* from markedly different bacterial species like *Bacillus pasteurii*, *Nitrosomonas europaea* and *Methylosinus trichosporium* OB3b (Figure 4A, B and C), while other bacterial cytochromes *c* like cytochrome *c*-555 from *Methylococcus capsulatus* (Bath) (see below) exhibit rhombic EPR spectra with *g*-values at 2.93, 2.28 and 1.49 (Figure 4D and Table 1).

**Table 1:** Ligand field and g-tensor values for the EPR signal from the *c*-type cytochromes (EPR spectra shown in Figure 4).

	g_max_	g_mid_	g_min_	V/ξ	Δ/ξ	V/Δ	Ref
*B. pasteurii c*-553	3.36	1.94	0.98	1.00	3.18	0.31	[44]
*N. europaea c*-552	3.34	1.87	1.17	1.09	4.45	0.24	[44]
*M. trichosporium* OB3b *c*-554	3.4	1.8	1.1	0.99	4.57	0.22	This work
*M. capsulatus* (Bath) *c*-555	2.93	2.28	1.49	1.87	3.06	0.61	This work

The line width of the two lower *g*-components (at higher magnetic field) in the HALS EPR signals is very broad and can not be accurately assigned from the spectrum. Simulations of the EPR signal shows that the spectrum is best fit with *g*_y_  = 1.8 (Figure 4E). The *g*_x_-value was too broad for any meaningful simulation, and was calculated to be 1.1 using the equation *g*_z_^2^+ *g*_y_^2^+ *g*_x_^2^ = 16 [40]. Using the equations of Taylor the crystal field parameters were calculated to be V/ξ  = 0.994, Δ/ξ  = 4.57. This gives the rhombicity V/Δ  = 0.22 and a^2^+ b^2^+ c^2^  = 1.0006, which is in the acceptable range demanded by this analysis [41]. These are refined values compared to what we previously reported [42], still fitting the observed trend for the *c*-type cytochromes with His/Met ligation that exhibit a ferric HALS EPR signal.

**Figure 5:**
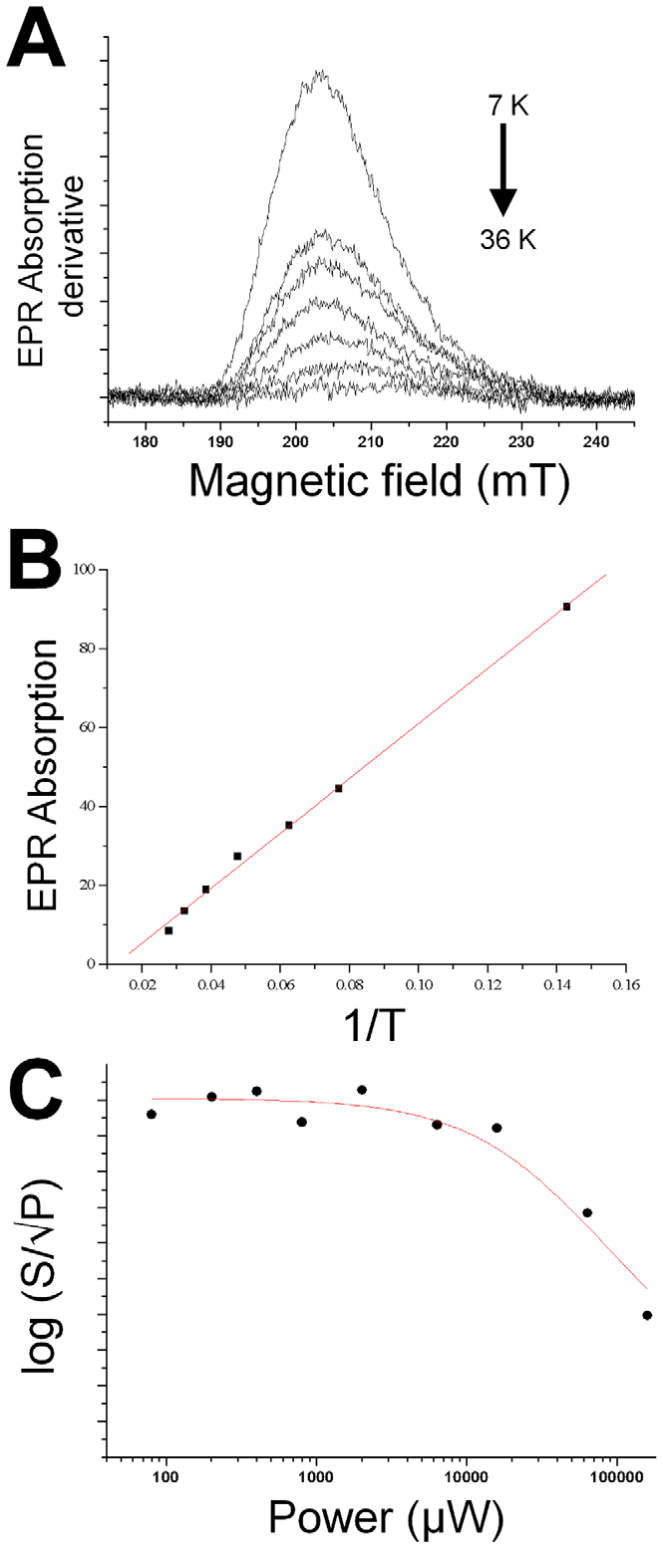
Temperature and microwave-power behaviour of the EPR signal of cytochrome *c*-554 from *Methylosinus trichosporium* OB3b. A. Temperature dependence of the HALS EPR spectrum. The spectra were recorded at 800 µW and the temperatures were 7, 13, 16, 21, 26, 31 and 36 respectively. B. The double integral of g-max plotted against the inverse temperature (1/T). The line represents the linear fit according to the Curie-law. C. Power saturation of the EPR spectrum. S equals the double integral of g-max, and P is the microwave power. The line is a simulated power saturation behaviour assuming a b-value of 1, indicating a P_½_ value of 44 mW. The spectra were recorded in perpendicular mode with modulation amplitude 1 mT, modulation frequency 100 kHz, sweep time 168 s, time constant 82 ms and 4×10^4^ gain.

At 10 K this EPR signal is difficult to saturate, revealed by a relatively high microwave power saturation value (P_½_). The P_½_ value was determined for the HALS EPR signal from cytochrome *c*-554 from *Methylosinus trichosporium* OB3b using the equation [43]:
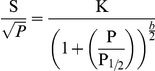
Where **S** denotes the double integral of the EPR signal, **K** is a normalisation factor, **P** is the microwave power, ***b*** is a component relating to the type of relaxation and **P_½_** is the microwave power at half saturation. The b factor can vary between 1 for a completely inhomogeneous relaxation, and 3 for a completely homogenous relaxation. The microwave power saturation behaviour of *Methylosinus trichosporium* OB3b cytochrome *c*-554 was simulated with a *b* factor of 1 and indicates a P**_½_** value of 44 mW±9 mW (Figure 5C). All the studied *c*-type cytochromes that exhibit HALS EPR spectra show P_½_ values above 40 mW at 10 K [44], [45], indicating that high saturation midpoint may be a general feature of the HALS EPR signal.

EPR spectra of the “*g*-max” signal were recorded at seven different temperatures ranging from 7 K to 36 K under non-saturating conditions (microwave power 0.8 mW) with all other instrumental parameters kept constant (Figure 5A). At 36 K, the EPR signal was barely visible. This is similar to the temperature behaviour of the other HALS EPR signals studied, and consistent with the former notion that these signals are not readily observed above 20 K [39], [44]. Figure 5B shows how the double integral of the EPR signal varies with the inverse absolute temperature. The linear behaviour shows that the magnetisation follows the Curie law in this temperature interval. This implies that the unpaired electron in the ferric cytochrome behaves like an ideal spin-½ paramagnet in the temperature range 7 K to 36 K, hence it is not influenced by neighbouring paramagnetic species.

### Cytochrome *c*-555 from *Methylococcus capsulatus* (Bath)

Frozen cells of the methane oxidizing gammaproteobacterium *Methylococcus capsulatus* (Bath) was received as a generous gift from DB Lab A/S in Odense, Denmark [46]. The same purification protocol as was used for cytochrome *c*-554 from *Methylosinus trichosporium* OB3b was used to purify a *c*-type protein from this bacterium. This *c*-type cytochrome has a molecular weight of 11120 Da as determined by MALDI-TOF MS. The first 14 amino acids were sequenced using Edman degradation and showed full identity with the cytochrome *c*-555 previously reported by Ambler et al. [47]. This cytochrome exhibits a rhombic EPR signal (Figure 4D, 4F and Table 1) where the crystal field parameters were calculated to be V/ξ  = 1.87 and Δ/ξ  = 3.06. This gives the rhombicity V/Δ  = 0.61 and a^2^+ b^2^+ c^2^ = 1.003.

## Discussion

HALS EPR signals can occur both in Histidine/Methionine ligated hemes like in cytochromes *c*_2_, and in bis-Histidine ligated hemes. In bis-Histidine ligated hemes, a relation between the geometry of the histidines and the different EPR spectra has been found [39], [48]. A similar structural basis for this signal in His/Met coordinated hemes is not understood [20], [21], [40], [42], [44], [45], [49]. The derived ligand field parameters for cytochrome *c*-554 from *Methylosinus trichosporium* Ob3b is V/ξ  = 0.99 and Δ/ξ  = 4.57, and a rhombicity V/Δ  = 0.22. This is similar to what we find in other *c*-type cytochromes (Table 1) where we observe that proteins exhibiting HALS EPR signals have V/ξ around 1.0 and a rhombicity lower than 1/3.

Six coordinated *c*-type cytochromes are electron transfer proteins, but the biological role of cytochrome *c*-554 from *Methylosinus trichosporium* Ob3b has not been elucidated. The homologues of cytochrome *c*-554 in the group cytochrome *c*_2_ is known to mediate electron transfer between *bc*_1_ complexes and cytochrome *c* oxidases during aerobic growth [19]. A similar role is possible for this cytochrome, as the high theoretical pI suggests a weak membrane association and the signal sequence suggests a periplasmic localisation, which is a common localisation for cytochromes *c* [50], or it might be involved in the metabolism of methane. The genome of *Methylosinus trichosporium* OB3b contains several (around 10) putative *c*-type cytochromes, but only a few is mentioned in the literature [51]. The homology with mitochondrial cytochrome *c* is not unreasonable as the mitochondrion is believed to originate from another alphaproteobacterium [52].

Cytochrome *c*-554 belongs to the class of cytochrome *c*_2_. The homologous mitochondrial cytochrome *c* has a rhombic EPR spectrum with *g*-values of 3.06, 2.24 and 1.24 [53] and the related membrane interacting cytochrome *c*_2_ from *Rhodobacter sphaeroides* has *g*-values of 3.29, 2.06 and 1.14 at neutral pH [54]. Both of these have known crystal structure [55]. Interestingly, the axial methionine ligand in the latter structure can be lost, as also suggested for horse heart cytochrome *c* at high pH [56]. This indicates, together with the fluxionality of the methionine ligand in *Nitrosomonas europaea* cytochrome *c*-552 that the methionine is loosely coordinated to the iron. The *g*-values of cytochrome *c*-554 from *Methylosinus trichosporium* OB3b is clearly HALS, and represent the high end of the *g*_max_ range observed for the small soluble cytochromes c [42]. The mitochondrial cytochrome *c* is found in low *g*_max_ end of this range, and cytochrome *c*_2_ from *Rhodobacter sphaeroides* is in the middle of this range. The homology with these membrane interacting cytochromes further strengthens the idea that cytochrome *c*-554 is membrane associated, and might have a weakly coordinated axial methionine. This study further indicate that subtle differences in the His/ Met ligated heme proteins can modulate the low-spin EPR signal [42], [45]. This is in agreement with preliminary, unpublished crystallographic studies, by Kara Bren's and K.Kristoffer Andersson's laboratories, on the *Nitrosomonas europaea* cytochrome *c*-552 mutant without the HALS EPR signal.
